# What Is Cholera?

**Published:** 1893-05

**Authors:** William Watson Hall


HALL'S
JOURNAL OF HEALTH

TRUTH DEMANDS NO SACRIFICE; ERROR CAN MAKE NONE.

VOL. XL. MAY, 1893. NO. 5.

### WHAT IS CHOLERA?

Cholera is the exaggeration of intestinal vermicular motion. This definition, explained in language less professional, would do more good than all the popular recipes for the cure of cholera ever published, because it expresses the inherent nature of cholera and suggests the principle of cure in its early stage, to the most unreflecting mind. The public is none the better, or wiser, or safer, for one of all the ten thousand "cures" for cholera proclaimed in the public prints, with a confidence which itself is a sufficient guarantee that however well informed the authors may be in other matters, as regards cholera itself they are criminally ignorant; for no man has a right to address the public on any subject connected with its general health unless he understands that subject in its broadest sense, practically as well as theoretically. A "live" cheese, or a cup of fishing worms may give an idea of the motion of the intestines in ordinary health.

The human gut is a hollow, flexible tube, between thirty and forty feet long; but, in order to be contained within the body, it is, to save space, arranged as a sailor would a coil of rope; forever moving in health, moving too much in some diseases, too little in others. To regulate this motion is the first object of the physician in every disease.

In headaches, bilious affections, costiveness, and the like, this great coiled-up intestine, usually called "the bowels," is "torpid," and the medicines are given to wake it up, and what that does cures the man. Costiveness is the foundation, that is, one of the first beginnings, or it is the attendant of every disease known to man, in some stage or other of its progress. But the human body is made in such a manner that a single step cannot be taken without tending to move the intestines ; thus it is, in the main, that those who move about on their feet a great deal have the least sickness, and, on the other hand, those who sit a great deal, and hence move about but little, never have sound health ; it is an impossibility, it is a rule to which I have never known an exception. Cholera being a disease in which the bowels move too much, the object should be to lessen that motion ; and, as every step a man takes, increases intestinal motion, the very first thing to be done in a case of cholera, is to secure quietude. It requires but a small amount of intelligence to put these ideas together, and if they could only be burnt in every heart, this fearful scourge would be robbed of myriads of its victims.

There can be no cure for cholera without quietude, the quietude of lying on the back. The physician who understands his calling is always on the lookout for the instincts of nature ; and he who follows them most, and interferes with them least, is the one who is more successful. They are worth more to him than all the rigmarole stories which real or imaginary invalids pour in upon the physician's ear with such facile volubility.

If, for example, a physician is called to a speechless patient, a stranger, about whom no one can give any information, he knows if the breathing is long, heavy and measured, that the brain is in danger ; if he breathes quick from the upper part of the chest, the abdomen needs attention ; or if the abdomen itself mainly moves in respiration, the lungs are suffering.

In violent cases of inflammation of the bowels, the patient shrinks involuntarily from any approach to that part of his person. These are the instincts of nature, and are invaluable guides in the treatment of disease. Applying this principle to cholera, or even common diarrhœa when the bowels do not act more than three or four times a day ; the patient feels such an unwillingness to motion that he even rises from his seat with the most unconquerable reluctance ; and when he has from any cause been moving about considerably, the first moment of taking a comfortable seat is perfectly delicious, and he feels as if he could almost stay there always.

The whole animal creation is subject to disease, and the fewest number, comparatively speaking, die of sickness ; instinct is their only physician. Perfect quietude, then, on the back, is the first, the imperative, the essential step towards the cure of any case of cholera. To this art may lend her aid towards making that quietude more perfect, by binding a cloth around the belly pretty firmly. This acts beneficially in diminishing the room within the abdomen for motion ; a man may be so pressed in a crowd as not to be able to stir. This bandage should be about a foot broad and long enough to be doubled over the belly ; pieces of tape should be sewn to one end of the flannel, and a corresponding number to another part, being safer and more effective fastening than pins. If this cloth is of stout woolen flannel it has two additional advantages, its roughness irritates the spine and draws the blood to the surface from the interior and by its clammy condition of the skin which takes place in the last stages of cholera. Facts confirm this. When the Asiatic scourge first broke out among the German soldiery immense numbers perished ; but an imperative order was issued in the hottest weather, that each soldier wear a stout woolen flannel abdominal compress, and immediately the fatality diminished of common looseness of bowels, he will generally find the most grateful and instantaneous relief. The second indication of instinct is to quench the thirst.

When the disease now called cholera first made its appearance in the United States, in 1832, it was generally believed that the drinking of cold water soon after calomel was taken, would certainly cause salivation ; and, as calomel was usually given, cold water was strictly interdicted. Some of the most heart-rending appeals I have ever noticed were for water, water ! I have seen the patient with deathly eagerness mouth the finger ends of the nurse for the sake of the drop or two of cold water there while washing the face. There are two ways of quenching this thirst, cold water and ice. Cold water often causes a sense of fullness or oppression, and not always satisfying ; at other times the stomach is so very irritable that it is ejected in a moment. Ice does not give that unpleasant fullness, nor does it increase the thirst, as cold water sometimes does, while the quantity required is very much reduced.

A CASE.

Some years ago I was violently attacked with cholera symptoms in a railroad car. The prominent symptoms were a continuous looseness of the most exhausting character, a deathly faintness and sickness, a drenching perspiration, an overpowering debility, and a pain as if the whole intestines were wrung together with strong hands, as washerwomen wring out clothing, Not being willing to take medicine, at least for a while, and no ice being presently obtainable, at the first stopping place I ate ice cream, or rather endeavored to swallow it before it could melt. I ate large quantities of it continually, until the thirst was entirely abated. The bowels acted but once or twice after I began to use it. I fell asleep, and next morning was at my office as usual, although I was feeble for some days. This may not have been an actual case of Asiatic cholera, although it was prevalent in the city at that time ; but it was sufficiently near it to require some attention, and this is the main object of these articles, to wit : attention to the first symptoms of cholera when it prevails.

According to my experience, there is only one objection to the ice cream treatment, and that is, you must swallow it without tasting how good it is ; it must be conveyed into the stomach as near an icy state as possible. The second step then, in the treatment of an attack of cholera, is to quench the thirst by keeping a plate of ice beside you, broken up in small pieces, so that they may be swallowed whole, as far as practicable ; keep on chewing and swallowing the ice until the thirst is most perfectly satisfied.

PRACTICAL RESULTS.

The first step, then, to be taken when cholera prevails and its symptoms are present is to lie down on a bed. 2d. Bind the abdomen tightly with woolen flannel. 3d. Swallow pellets of ice to the fullest extent practicable. 4th. Send for an established, resident regular physician. Touch not an atom of the thousand things proposed by brains as "simple" as the remedies are represented to be, but wait quietly and patiently wait until the arrival of your medical attendant.

But many of my readers may be in a condition, by distance or otherwise, where it is not possible to obtain a physician for several hours, and where such a delay might prove fatal. Under such circumstances, obtain ten grains of calomel and make it into a pill with a few drops of cold water ; dry it a little by the fire or in the sun and swallow it down. If the passages do not cease within two hours, then swallow two more such pills and continue to swallow two more at the end of each two hours until the bowels cease to give their light colored passages, or until the physician arrives.

In many bad cases of cholera, the stomach will retain nothing fluid or solid, cold water itself being instantly returned. A calomel pill is almost as heavy as a bullet ; it sinks instantly to the bottom of the stomach and no power of vomiting can return it.

It would answer just as well to swallow it in powder ; but the same medium which would hold it in suspension while going down, would do the same while coming up. The first object of a calomel pill in cholera is to stop the passages from the bowels. The treatment is effectual, it arrests the passages within two hours ; and in any time from four to twelve hours after being taken it effects the bowels actively, and the passages are changed from a watery thinness to a mushy thickness or consistency, and instead of being the color of rice water or of a milk and water mixture, they are brown or yellow, or green or dark, or black as ink according to the violence of the attack. Never take any thing to work off calomel, if there is any passage within ten hours after it is taken ; but if there is no passage from the bowels within ten, or at most twelve hours after taking calomel, then take an injection of common water, cool or tepid. Eating ice or drinking cold water after a dose of calomel, facilitates its operation and never can have any effect whatever towards causing salivation ; that is caused by there being no action from the bowels, as a consequence of the calomel, sooner than ten or twelve hours after it has been swallowed.

My own views, as a result of two and three years baffling in the midst of prevalent cholera, are, that when calomel fails to cure it, everything else will fail, and that it will cure every curable case.

The cure of this scourge depends upon the earliness with which the means are used. It can be said with less limitation than of all other diseases together, that cholera more certainly kills if let alone, and is certainly cured if early attended to. What, then, is the earliest and almost universal symptom of approaching cholera ?

I have never seen it named in print as such. During my personal experience amidst the scourge when it last visited this country, I could tell in my own office, without reading a paper, or seeing or speaking to a single person, the comparative prevalence of the disease from day to day by the sensation which I will name and I hope to the benefit of thousands, and perhaps not a single reader will fail to respond to the statement from his own experience.

The bowels may be acting but once or less than once in twenty-four hours, the appetite may be good, and the sleep may be sound ; but there is an unpleasant sensation in the belly, I do not, for the sake of delicacy, say "*stomach*," for it is a perversion of terms ; it is not in the stomach, nor do I call it the abdomen.

Many persons don't know what abdomen means.

Thousands have such good health that they have no "realizing sense" of being the owners of such "*apparati*," or "*usses*," as the reader may fancy, and it is a great pleasure to me to write in such a manner that I know my reader will understand me perfectly, without having the headache.

Speaking then of that sensation of uneasiness, without acute pain, in the region named, it comes on more decidedly after an evacuation of the bowels.

In health this act is followed by a sense of relief or comfortableness, but when the cholera influence is in the atmosphere, even a regular passage is followed by something of this sort, but more and more decided after each action over one in twenty-four hours. The feeling is not all; there is a sense of tiredness or weariness which inclines you to take a seat; to sit down, may be to bend over a little or to curl up, if on a bed. This sensation is coming cholera, and if heeded when first noticed would save annually, thousands. The patient should remain on the bed until he felt as if he wanted to get up and as if it would be pleasurable to walk about. While observing this quiet and while swallowing lumps of ice, nothing should be eaten until there is a decided appetite, and what is eaten should be farina or arrow-root, or tapioca or corn-starch, or what is better than all, a mush made of rice flour, or, if preferred, common rice parched as coffee, and then boiled, as rice is usually for the table, about twelve minutes, then strain the liquid from the rice; return the rice to the stew pan and let it steam about a quarter of an hour, a short distance from the fire; it will then be done, the grains will be separate; it may then be eaten with a little butter at intervals of five hours. There can be no doubt that thousands upon thousands have died of cholera who might now be living had they done nothing but observed strict bodily quietness under the promptings of nature, the greatest and the best physician.

WILLIAM WATSON HALL, M. D.

[To be continued.]

